# A predictive model for damp risk in english housing with explainable AI

**DOI:** 10.1038/s41598-025-96396-7

**Published:** 2025-04-12

**Authors:** Gulala Aziz, Adam Hardy

**Affiliations:** Leeds Sustainability Institute, Leeds Beckett University, Headingley Campus, Churchwood House, G02, Leeds, UK

**Keywords:** Machine learning, Damp management, English housing, Damp home characteristics, SHAP analysis, Causal analysis, Engineering, Civil engineering, Energy infrastructure

## Abstract

Damp in residential buildings poses risks to indoor air quality, occupant health, and structural integrity, and affects up to 27% of homes in the England. This study develops a predictive model for damp risk, using 2,073 inspection records from a housing association across 125 local authorities. Homes were labelled as damp (1,630) or non-damp (443), with data supplemented by national Energy Performance Certificate (EPC) records, incorporating building characteristics and energy efficiency indicators. To evaluate model performance, both a balanced dataset (869 homes, 426 damp, 443 non-damp) and a larger imbalanced dataset (2,073 homes) were used. Seven machine learning algorithms were deployed, with the best-performing model achieving 0.636 accuracy on balanced data and 0.793 on imbalanced data. SHAP (SHapley Additive exPlanations) analysis identified heating cost, energy consumption, and wall energy efficiency as the strongest predictors of damp. Statistical tests and causal analysis were applied to interpret SHAP results, offering insights into potential damp risk and mitigations. The findings suggest that machine learning can support early identification of homes likely to develop damp, helping housing managers prioritise interventions before damp issues escalate.

**Supplementary Information:**

The online version contains supplementary material available at 10.1038/s41598-025-96396-7.

## Introduction

Damp in residential buildings is a global issue with significant, long-term consequences. Referring to the presence of excess or unwanted moisture^1^, damp is usually caused by condensation, leaks, or moisture which has penetrated the envelope of a property^2–5^. Once it sets in, it can create a self-perpetuating cycle: the presence of excess moisture can weaken insulation and increase a home’s thermal conductivity^6^, making spaces colder. In turn, colder interiors tend to have higher relative humidity, elevating the risk of condensation and exacerbating damp issues even further. As a result, damp can be difficult to eradicate, yet it carries extensive ramifications for both buildings, and for residents who have to live with damp.

One of damp’s most serious consequences is its impact on occupant health. In the UK, The All Party Parliamentary Group on Healthy Homes and Buildings (2018) highlights that poor indoor air quality (IAQ) in damp homes causes the loss of 204,000 healthy life years annually^7^. The impact of damp homes is also more pronounced for vulnerable groups such as children^1,8^. They double the risk of respiratory illnesses, contribute to 10 to 15% of new childhood asthma cases, and worsen lung function. Education further suffers, with UK children missing 80% more school days due to damp-related illnesses than the European average, totalling 1.7 million school days missed due to illnesses associated with damp and mould^1,8^. The recent death of a two-year-old, who died from a respiratory infection caused by exposure to mould in his home, tragically underscores the continuing severity of the issue^9^. This event has exposed gaps in the current housing management strategies and prompted the UK Government to call for more proactive approaches in addressing damp-related issues^9^.

Excessive moisture not only threatens occupant health but can also compromise the durability and value of the building fabric. Indeed, it is estimated that 70 to 80% of building damage results from excessive or trapped moisture^10,11^. These issues have spurned many advancements in damp research and construction techniques^12,13^, but damp remains a persistent problem, manifesting in various forms such as rising damp, penetrating damp, leaks, and condensation. Indeed, several modern developments such as increased airtightness in modern homes, overcrowding, fuel poverty, and evolving building usage patterns, are in many cases exacerbating these damp problems in residential properties^10^.

The Building Research Establishment (BRE)^14^ analysed the financial burden of Damp on the UK’s economy. In its 2021 report on the cost of poor housing, BRE estimated that the UK’s National Health Service spends approximately £1.4 billion annually treating individuals affected by poor housing conditions, with around £895 million of this directly linked to defects that expose residents to excess cold or damp. In addition to healthcare costs, BRE also emphasised the broader societal impacts, including increased care needs, reduced economic potential, diminished educational outcomes, and adverse mental health effects. Factoring in these additional societal costs, the overall cost to the economy from cold or damp housing is estimated at £15.4 billion per year.

Despite the negative impacts, damp remains poorly understood and ill-quantified. A UK Centre for Moisture (UKCMB) report cited estimates from the Energy Savings Trust that around a third of the UK population (over 8 million properties and 20 million people) report having mould in their homes^15^. However, this figure contrasts sharply with the 2017 English Housing Survey, which found that only 4% of homes were identified by surveyors as having damp or mould issues. Conversely, self-reported data from the same survey indicates that nearly 30% of households, or around 7 million homes, experienced problems with damp, condensation, or mould^16^. These discrepancies reflect challenges in accurately assessing damp in homes, with government estimates ranging from 4 to 27% of households (approximately 962,000 to 6.5 million homes), depending on the measurement method used^17^.

While damp can be hard to quantify, its prevalence certainly appears to have a socioeconomic element. A 2023 UK Government housing report^18^states that renters, whether in private or social housing, are more vulnerable to damp and mould compared to homeowners. The Energy Follow-Up Survey indicates that 41% of households in the lowest income quintile report damp and mould issues, compared to only 16–23% in the highest income quintiles^19^. Additionally, the UK Government’s Fuel Poverty Report reveals that 42% of households experiencing fuel poverty report damp, compared to 25% of those not in fuel poverty^20^. Certain types of households are also particularly vulnerable to living in damp homes, including younger (25 to 44 years), overcrowded (5 to 6 person households), and low-income households^16^. Ethnic disparities also play a significant role in the distribution of damp-related issues in the UK. Data from the English Housing Survey (2019) show that 10.4% of Black households report experiencing damp problems, compared to 3.8% of White households and 6.2% of Asian households^1,21^. These figures reflect broader inequalities in housing conditions and access to quality, affordable homes.

In a social-housing context, damp will most often be identified by the resident of a property. If this damp is reported, it will usually trigger a reactive physical inspection, after which a damp management plan will be devised. This strategy relies heavily on manual identification of damp problems, which is often slow and precludes opportunities for early intervention. The speed of this process underscores the need for a more forward-thinking, data-driven approach to managing damp in residential buildings.

One promising avenue for proactive damp management is the application of machine learning. Machine learning has been widely adopted in various fields such as healthcare, finance, energy management, and building performance prediction, offering powerful tools for data-driven decision-making^22,23^. In particular, machine learning models have been successfully used to predict building performance, energy consumption, and fault detection^23–26^. But, despite its growing applications, there is a notable lack of research on using machine learning for predicting damp issues in homes, particularly in a residential setting.

This gap represents an opportunity to apply machine learning techniques to a critical area of housing management. By utilizing machine learning models, it may be possible to analyse large datasets, including building characteristics and energy performance, to predict the likelihood of damp problems. This approach would enable housing associations and landlords to take preventive measures before damp escalates into a more serious and costly issue. Machine learning, therefore, has the potential to transform the way damp is managed by shifting from reactive inspections to predictive, data-driven planning. This study aims to address the gap in predictive models for damp by investigating the applications of machine learning algorithms to predict damp issues in residential homes in the UK. In doing so, the research will contribute to the growing field of machine learning applications in housing management and lays the groundwork for more proactive strategies to mitigate damp, ultimately improving housing quality and occupant health.

## Results

### Results of model performance and best model selection

This study evaluated model performance using a balanced dataset of 869 homes (426 damp, 443 non-damp) and an imbalanced dataset of 2,073 homes (1,630 damp, 443 non-damp). In the latter case, damp homes were overrepresented due to targeted damp inspections for those homes. The target variable was binary (“has damp” or “non-damp”), with the model trained on 13 building characteristics and energy efficiency variables (see Table 1 for full details). These training variables were obtained from Energy Performance Certificates (EPCs) and included factors related to energy efficiency, building structure, and building composition. The balanced dataset allowed fair representation of both categories, while the imbalanced dataset tested the algorithm on the imbalanced conditions which are more likely to be encountered in the real-world.

**Table 1:** Variables included within the model to predict a home’s risk of damp.

Variable name	Description
hasDamp	The **dependant variable** for the model. A TRUE/FALSE Indicator for whether a home has damp issues. This is the outcome variable that the model aims to predict.
Current Energy Efficiency	A numerical measure of the home’s current energy efficiency.
Property Type	A categorical variable describing the type of property (e.g., flat, house, bungalow).
Energy Consumption Current	A numerical variable indicating the EPC-predicted energy consumption of the home (kWh per year)
Heating Cost Current	A numerical variable representing the EPC-predicted annual heating cost (£ per year), calculated using modelled energy efficiency estimates under standardised conditions.
Total Floor Area	A numerical variable denoting the total floor area of the property (square meters).
Number of Habitable Rooms	A numerical variable indicating the number of habitable rooms in the home.
Number of Heated Rooms	A numerical variable specifying the number of rooms that are heated within the home.
Current Energy Rating	A categorical variable representing the current energy rating of the home (e.g., A, B, C, etc.).
Built Form	A categorical variable describing the building’s structure or form (e.g., detached, semi-detached, terraced).
Floor Description	A categorical variable providing details about the type of flooring or floor construction (e.g., solid, suspended).
Walls Description	A categorical variable detailing the type of walls or wall insulation (e.g., cavity wall, solid wall).
Walls Energy Efficiency	A categorical variable indicating the energy efficiency rating of the walls.
Construction Age Band	A categorical variable indicating the age band or period when the property was constructed (e.g., pre-1900, 1967–1975).

Each dataset (balanced or imbalanced) was divided into two parts: 70% of the data was allocated to the training set for the purpose of model development (which includes training and cross-validation), and the remaining 30% of the data was set aside as the test set. The test set, often referred to as the “blind test,” was never used during the training phase. This separate 30% test set was reserved to independently evaluate how well the model could generalize to new, unseen data.

Within the training set, 10-fold cross-validation was applied: in each fold, the model was trained on 90% of the data and validated on the remaining 10%. This approach provided an average performance estimate, improving reliability before final testing on unseen data. After cross-validation, models were tested on the blind test set. This final evaluation was performed separately for both balanced and imbalanced datasets to assess generalization and provide an unbiased measure of model performance.

The model’s performance in cross-validation was then compared to its performance on the blind test set. If the test results were significantly lower than cross-validation scores, it indicated overfitting, meaning the model performed well on familiar data but struggled with unseen cases. However, if cross-validation and test performance were similar, it suggested that the model generalized well to new data.

In both the training-validation and testing phases, models were evaluated using accuracy, precision, recall, F-measure, and AUC, each ranging from 0 to 1, where higher values indicate better performance. Accuracy measured overall correctness, with 1 representing perfect predictions and 0 indicating no correct classifications. Precision assessed how many homes predicted as damp were damp, minimizing false positives (i.e., non-damp homes incorrectly classified as damp). Recall measured how many actual damp homes were correctly identified, reducing false negatives (i.e., damp homes incorrectly classified as non-damp). The F-measure provided a single performance score by balancing precision and recall. The AUC evaluated how well the model distinguished between damp and non-damp homes, with values closer to 1 indicating stronger classification ability.

The comparison between average validation performance across 10 folds (Table 2) and blind test results (Table 3) shows that models trained on balanced data generalized better, as their test performance remained consistent with validation results across all metrics. In contrast, models trained on imbalanced data showed notable drops in recall and F-measure when tested on unseen data, suggesting they struggled to identify damp homes correctly. This issue was most severe in the decision trees and logistic regression, where recall dropped to near zero, indicating overfitting to the majority class. While the support vector machine (SVM) and random forest demonstrated better generalization on the balanced dataset, XGBoost and random forest performed relatively better than other models on the imbalanced dataset, though their recall still declined. Overall, models trained on balanced data avoided overfitting, whereas those trained on imbalanced data overfitted to majority class patterns, leading to poor generalization in real-world scenarios.

**Table 2:** Training and validation performance of machine learning algorithms on balanced and imbalanced datasets.

ML Algorithms	Data sets	Training and validation performance				
		Accuracy	Precision	Recall (Sensitivity)	F-measure	AUC
1. Neural Network	Balanced Data	0.616	0.621	0.655	0.638	0.615
	Imbalanced Data	0.783	0.488	0.199	0.283	0.789
2.Decision Tree	Balanced Data	0.580	0.570	0.607	0.588	0.545
	Imbalanced Data	0.758	0.301	0.051	0.087	0.535
3.XGBoost	Balanced Data	0.641	0.634	0.646	0.640	0.669
	Imbalanced Data	0.790	0.518	0.315	0.392	0.749
4.Random Forest	Balanced Data	0.606	0.594	0.736	0.658	0.653
	Imbalanced Data	0.800	0.577	0.263	0.362	0.746
5.Support Vector Machine (SVM)	Balanced Data	0.595	0.577	0.807	0.672	0.632
	Imbalanced Data	0.786	0.50	0.032	0.060	0.648
6.Logistic Regression	Balanced Data	0.585	0.588	0.649	0.617	0.634
	Imbalanced Data	0.785	0.50	0.006	0.012	0.654
7.Nearst Neighbours (KNN)	Balanced Data	0.598	0.595	0.694	0.640	0.645
	Imbalanced Data	0.780	0.473	0.199	0.280	0.677

**Table 3:** Performance metrics of machine learning algorithms on balanced and imbalanced datasets using blind test evaluation.

ML Algorithms	Data sets	Blind test performance				
		Accuracy	Precision	Recall (Sensitivity)	F-measure	AUC
1. Neural Network	Balanced Data	0.613	0.617	0.659	0.637	0.666
	Imbalanced Data	0.787	0.50	0.234	0.319	0.585
2.Decision Tree	Balanced Data	0.578	0.577	0.674	0.622	0.432
	Imbalanced Data	0.787	0	0	0	0.50
3.XGBoost	Balanced Data	0.585	0.592	0.628	0.610	0.604
	Imbalanced Data	0.787	0.50	0.280	0.359	0.701
4.Random Forest	Balanced Data	0.636	0.640	0.742	0.657	0.676
	Imbalanced Data	0.793	0.533	0.242	0.333	0.716
5.Support Vector Machine (SVM)	Balanced Data	0.656	0.619	0.863	0.721	0.656
	Imbalanced Data	0.784	0.40	0.030	0.056	0.645
6.Logistic Regression	Balanced Data	0.601	0.598	0.689	0.640	0.649
	Imbalanced Data	0.782	0.20	0.007	0.014	0.499
7.Nearst Neighbours (KNN)	Balanced Data	0.560	0.591	0.666	0.626	0.653
	Imbalanced Data	0.792	0.529	0.204	0.295	0.6

**Fig. 1:**
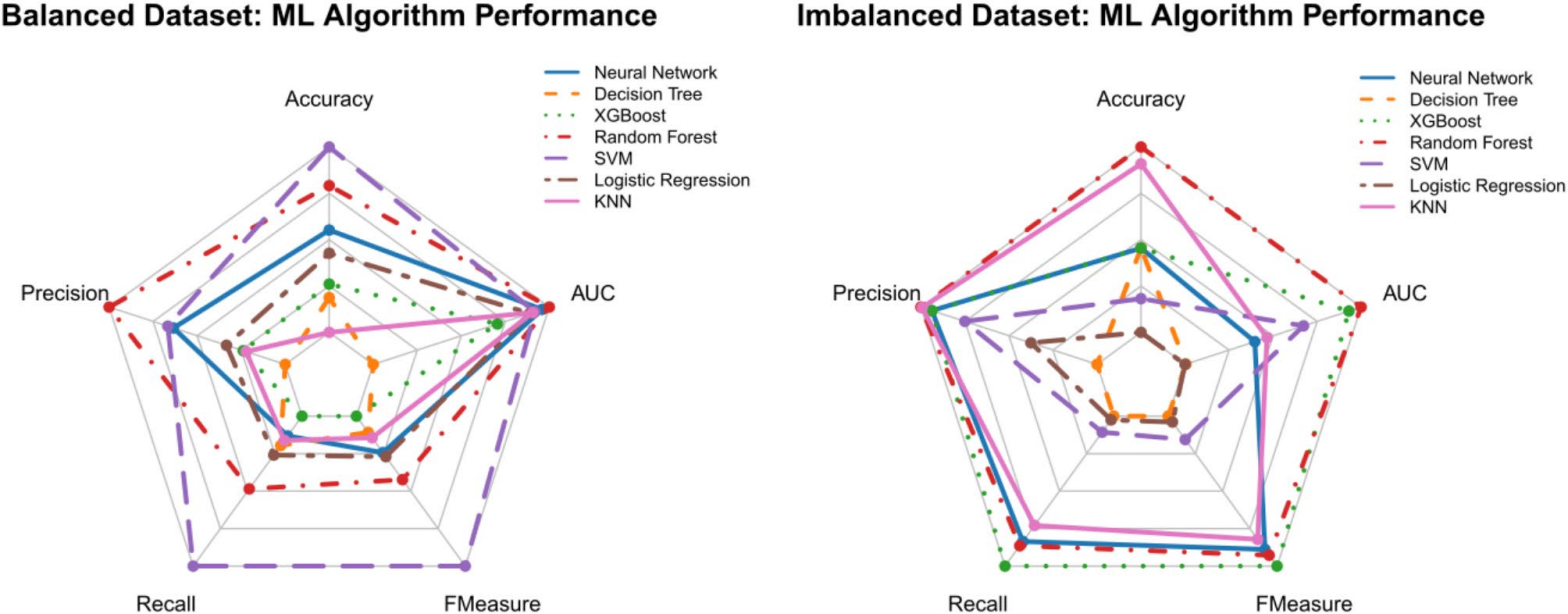
Comparison of Model Performance on Balanced and Imbalanced Datasets During the Blind Test Phase, Evaluating Accuracy, Precision, Recall, F-Measure, and AUC Across Seven Machine Learning Algorithms.

The results of the blind test (visualized in Fig. 1 and in Table 3) indicate that XGBoost and random forest were the most reliable models, maintaining a good balance between recall and AUC across datasets. A higher recall is preferable when the goal is to correctly identify damp homes, while AUC provides a more holistic assessment of classification performance. The random Forest demonstrated the highest AUC (0.716) on the imbalanced dataset, suggesting its effectiveness in differentiating between damp and non-damp homes in datasets with uneven class distributions. XGBoost followed closely with an AUC of 0.701, maintaining a stable balance between precision and recall. These results indicate that Random Forest showed a strong trade-off between recall, precision, and AUC across both datasets, making it a suitable choice for damp risk prediction in varying data conditions. XGBoost also performed well, particularly in scenarios where optimizing the precision-recall balance is a priority.

The SVM performed well in balanced data but struggled in imbalanced scenarios, while the decision tree and logistic regression were the least effective, particularly in identifying non-damp homes. The generally higher accuracy observed in imbalanced data must be interpreted cautiously, as it primarily reflects the model’s bias toward predicting the majority class rather than its true predictive capability.

The findings suggest that both random forests and XGBoost are strong candidate algorithms for predicting homes risk of damp from building characteristic data, especially if the dataset is imbalanced, as would likely be the case in a real-world application. This research therefore opens the possibility that housing associations can use such algorithms in a program of proactive maintenance, visiting homes with a high risk of damp and intervening before damp becomes a serious issue. However, such a program of maintenance would benefit from understanding why the predictive algorithm reached its decisions, and what causal pathways may exist which can be interrupted. In the following sections, an investigation into how and why the algorithms reached their predictions will be conducted. Given its strong performance across both datasets, the random forest was chosen for this analysis.

### Key factors in damp prediction

**Fig. 2:**
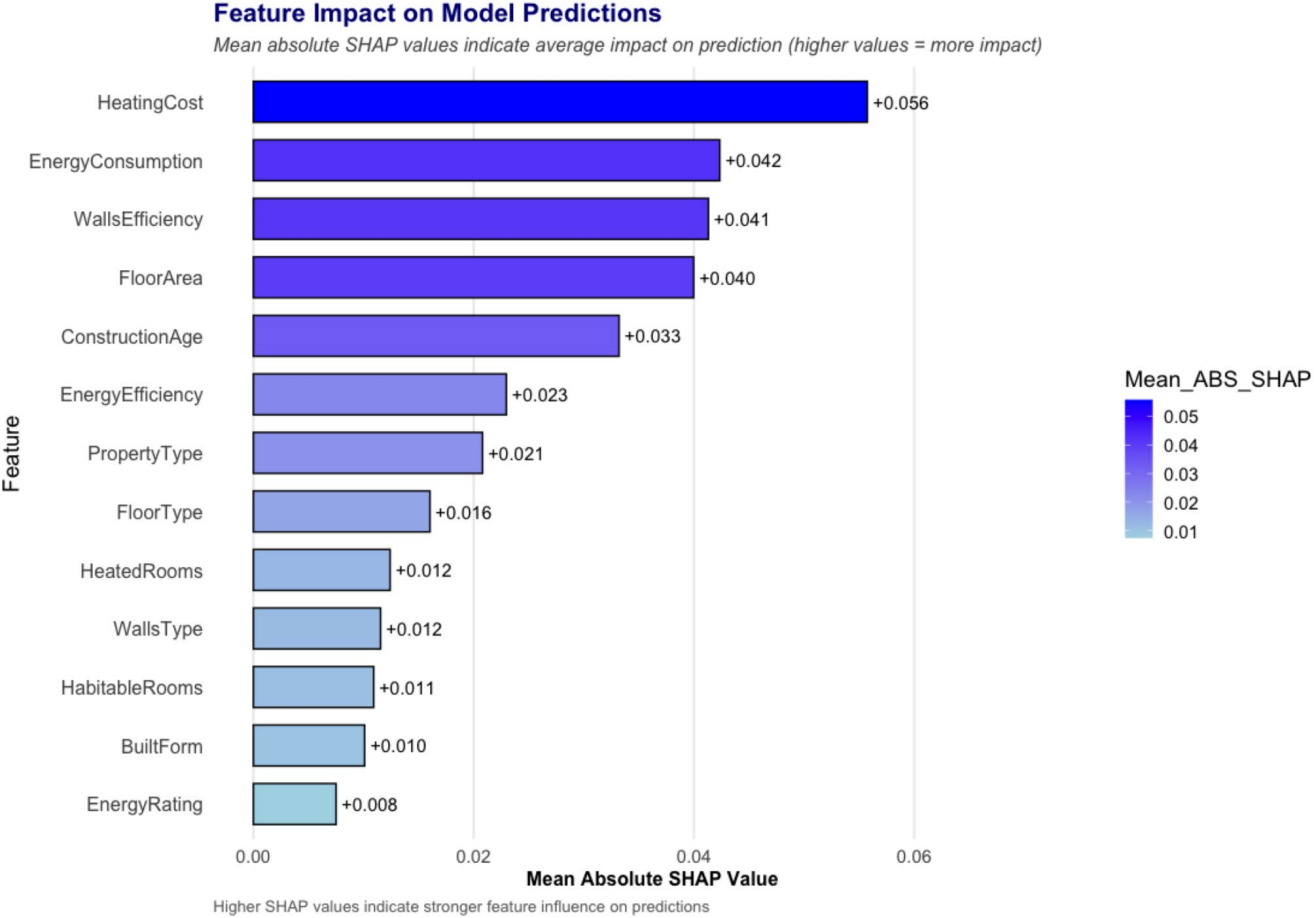
Feature Impact on Model Predictions: Analysis of Mean Absolute SHAP Values. Longer bars indicate a greater contribution to damp risk prediction.

**Fig. 3:**
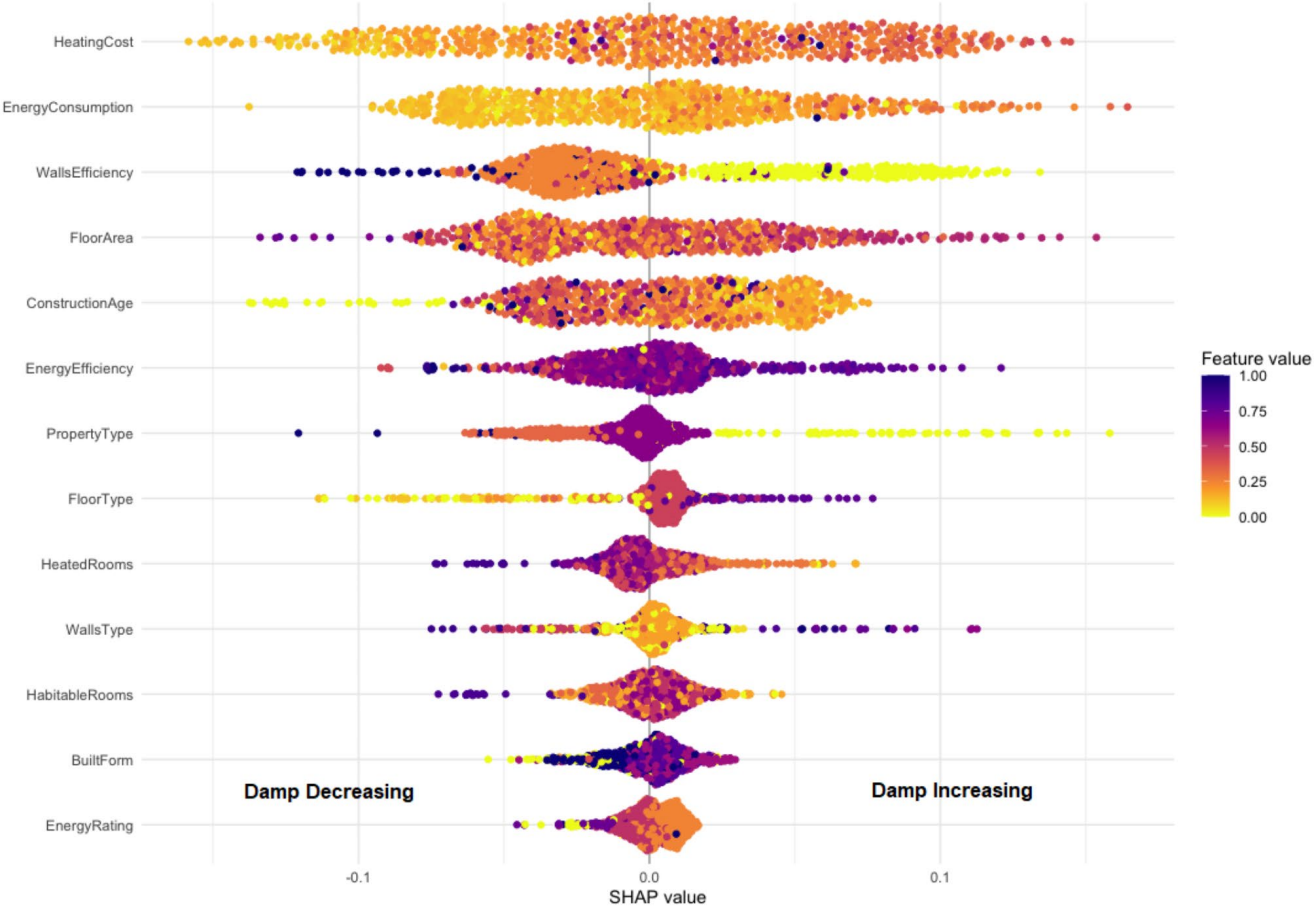
SHAP Beeswarm Plot illustrating the impact of each feature on the likelihood of damp issue.

To identify the key contributing variables influencing the risk of damp in homes, SHAP (SHapley Additive exPlanations) was conducted on the random forest, providing insights into how each variable contributes to the model’s output both globally and at an individual prediction level. Figure 2 shows the SHAP values calculated for each variable. The higher these SHAP values, the more impact the variable had on prediction. To investigate further how the algorithm reached its predictions, Fig. 3 shows a beeswarm plot for the input variables. Each point in this plot represents a single input from a home. Inputs with a negative SHAP value (to the left of the plot) contributed to a negative prediction (i.e., they suggested a home without damp). Conversely, inputs with a positive SHAP value (to the right of the plot) contributed to a positive prediction, and thus suggested the home had the presence of damp. The variables are further colour coded to indicate if they took a high or low absolute value. Taking “Heating Cost” as an example, higher values for this variable tended to result in positive predictions for damp in a home.

The most important variables are studied in more detail below:

### *Heating cost and energy consumption*

It is apparent from Fig. 2 that “Heating Cost” emerged as the most critical input variable influencing damp risk predictions, followed by Energy Consumption. Figure 3 further suggests that homes with high heating costs and energy consumption tend to result in positive predictions for damp. However, it is important to note that these variables do not represent actual energy consumption incurred by the household, but rather the modelled energy demand, calculated by the EPC, for maintaining a standard indoor temperature. This means that a property with low energy efficiency will have a high heating cost according to the EPC, regardless of how much energy is actually consumed by the occupants. High modelled heating costs typically indicate poor insulation or inefficient heating systems. As the data in this study comes from social housing, it’s likely that affordability constraints cause some homes with high modelled heating to underheat. This would be creating colder indoor environments and higher relative humidity- conditions that are conducive to damp formation. It is also worth mentioning that heating cost and energy consumption are closely related in an EPC. The former traces energy consumed on heating, whereas the latter traces energy consumed on heating, hot water, and lighting. It is therefore not surprising that the variables display similar importance and patterns in Fig. 3.

A Mann-Whitney U test applied to the heating cost of the damp, and non-damp homes revealed a p-value of 1.54 × 10⁻⁶, confirming a statistical difference between the two groups. The tests show that homes with higher heating costs are significantly more likely to be classified as damp. A second Mann-Whitney U test on SHAP values of heating cost produced a p-value of 0.0127, indicating that heating cost is not only different across damp classifications but also plays a significant role in predicting damp risk within the model.

Given the likely relationship between heating cost and other building variables such as wall energy efficiency and floor area, causal mediation analysis was conducted to determine whether heating cost is simply a mediator for the effect of wall energy efficiency and floor area. Using nonparametric bootstrapping with 1,000 simulations^27^, revealed that a portion of the effect of poor insulation on damp risk operates through increased heating costs. Specifically, 15.7% of the effect of wall energy efficiency on damp was mediated by heating cost (*p* < 0.001), meaning that poor insulation leads to higher heating costs, which in turn increase damp risk. However, the majority of the effect of wall energy efficiency on damp (84.3%) remained direct. This suggests that factors beyond just the mediator role of heating cost, such as thermal bridging or direct moisture penetration, also contribute significantly to damp risk. In contrast, floor area did not show a significant direct or mediated effect on damp risk, indicating that home size alone does not inherently drive heating costs or damp formation.

#### *Wall energy efficiency*

**Fig. 4:**
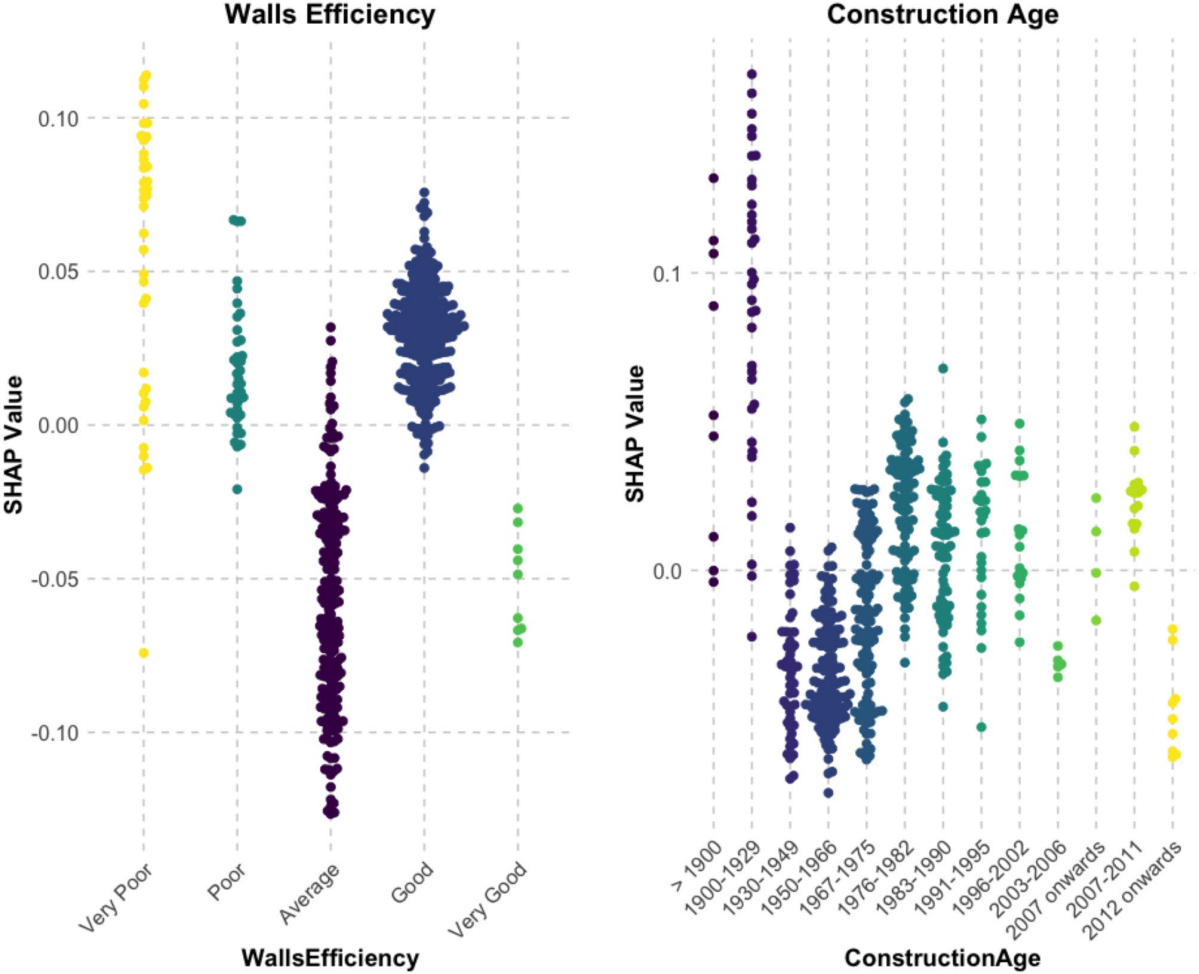
SHAP Value Distributions for Wall Efficiency and Construction Age: Positive values indicate higher damp risk, negative values indicate lower risk, and larger magnitudes reflect stronger predictive influence.

SHAP values (Fig. 2) highlight that wall efficiency is among the most influential predictors of damp risk. The dataset categorizes wall efficiency into five levels: “Very Poor,” “Poor,” “Average,” “Good,” and “Very Good”. The colour coding in Fig. 3 is expanded on in Fig. 4 which further illustrates that homes classified as “Very Poor” or “Poor” consistently show positive SHAP values, indicating a higher likelihood of damp. The data shows that the “Very Poor” wall energy efficiency group contains 84% solid wall as-built with no insulation, while the “Poor” group contains 84% cavity wall as-built with no insulation. This pattern aligns with well-established building physics principles, where poor insulation leads to greater heat loss, colder internal surfaces, and increased condensation risk-conditions that promote damp formation. In contrast, homes classified as having “Very Good” wall efficiency (67% as-built cavity wall and 33% Timber frame as-built insulated) tend to have negative SHAP values, suggesting a lower risk of damp due to effective insulation that stabilizes indoor temperatures and reduces moisture accumulation.

To statistically assess the relationship between Wall Energy Efficiency and damp occurrence, both a Chi-Square test of independence and logistic regression analysis were applied. The Chi-Square test (*p* = 2.06 × 10⁻⁹) confirmed a significant association between wall efficiency levels and damp status, indicating that insulation quality is not randomly distributed across damp and non-damp homes. This test is useful for detecting whether a relationship exists between two categorical variables but does not account for potential confounding factors that might influence damp occurrence.

To provide a more precise estimate of the impact of wall insulation while considering other influencing factors, a logistic regression analysis was conducted. This approach models the probability of a home experiencing damp while controlling for Floor Area, Heating Cost, Energy Consumption, and Construction Age. Controlling for these factors means that the analysis isolates the specific contribution of Wall Energy Efficiency to damp risk while holding the other variables constant. This ensures that any observed effect is not simply due to larger homes, higher heating costs, increased energy consumption, or older buildings, which are also known to influence damp occurrence. The logistic regression results showed that homes with “Poor” (*p* = 0.015) and “Very Poor” (*p* = 0.021) wall efficiency were significantly more likely to experience damp, reinforcing the well-established link between inadequate insulation and increased damp in homes.

Despite expectations that improved insulation would reduce damp risk, homes with “Good” wall efficiency show a significant positive association with damp risk, as reflected in their higher SHAP values in Fig. 4. This anomaly may be explained by the wall composition of “Good” energy efficiency homes in the dataset, where 59% have retrofitted cavity walls, 18% have as-built insulated cavity walls, and 6.6% have system-built walls. These three wall types can be vulnerable to damp if ventilation is insufficient, insulation is improperly installed, or construction defects compromise moisture resistance. Retrofitted cavity walls, if not properly installed or combined with ventilation improvements, may trap moisture rather than mitigate damp risk^28^. As-built insulated cavity walls can be compromised by installation defects such as incomplete filling and mortar obstructions, creating cold spots and moisture ingress, as documented in the BRE Good Repair Guide^29^. Finally, the system-built homes are predominantly small flats in the data, which research associates with higher humidity and condensation risk when ventilation is inadequate^30^.

#### *Floor area*

Floor area emerged as another contributing factor in damp risk prediction, following heating cost, energy consumption, and wall energy efficiency (Fig. 2). The data suggests that larger homes tend to have a lower likelihood of damp, as seen in Fig. 3, where floor area values for damp homes were generally lower than those for non-damp homes. However, while this trend was evident, the distinction was less pronounced compared to other key variables.

To further examine the role of floor area in damp risk, multiple statistical tests were conducted. A Mann-Whitney U test confirmed significant differences between the floor area of damp and non-damp homes (*p* = 0.0055). The distribution confirmed that larger homes were more frequently classified as non-damp, while smaller homes showed a higher likelihood of damp. Likewise, a U test on the SHAP values yielded a p-value of < 2.2e-16, confirming that floor area was significant in the model. However, further analysis using the Overlap Coefficient (OVL = 0.79) suggested that while floor area influences damp risk, the distributions of damp and non-damp homes substantially overlap, implying that home size has a complex relationship with damp risk, potentially mediated by other variables.

To explore how floor area and insulation quality interact, an ANOVA test (*p* < 0.001) was conducted, confirming a significant relationship between home size and wall energy efficiency. However, post-hoc testing using Tukey’s test revealed that the key driver of this relationship was a significant difference between homes classified as having “Good” and “Average” wall efficiency (*p* < 0.001), with better-insulated homes tending to be smaller. Notably, no significant difference was found between homes with Poor insulation and other categories (*p* > 0.05), suggesting that both small and large homes can have inadequate insulation. These findings indicate that while larger homes may generally have lower damp risk, insulation effectiveness and other structural factors play a more decisive role in determining moisture-related issues.

Overall, while floor area appears to play a role in damp risk, it should not be viewed in isolation. The findings suggest that a more holistic assessment considering insulation quality, ventilation, and other structural factors is needed to understand why some homes are more vulnerable to damp than others.

**Fig. 5:**
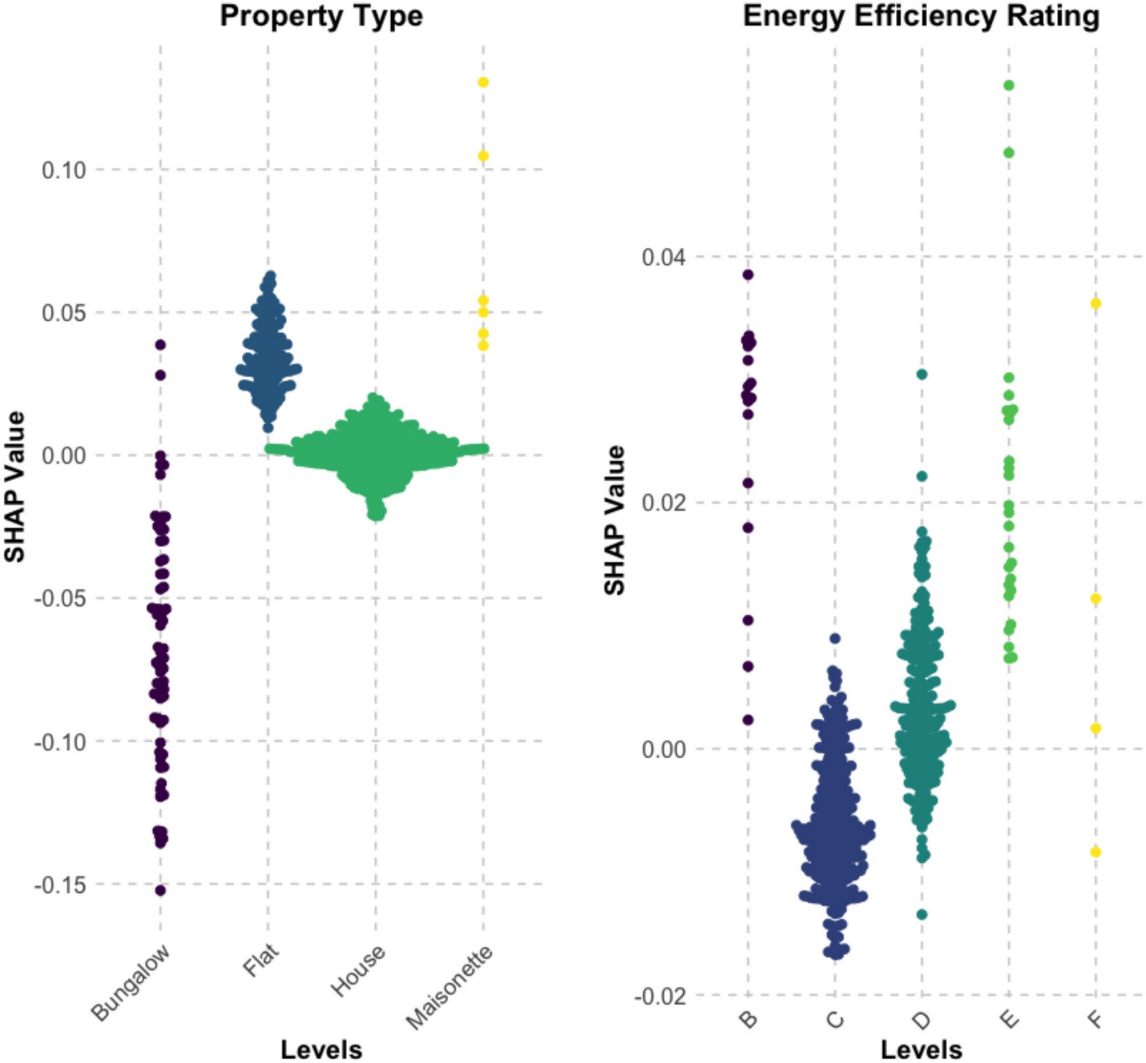
SHAP Value Distributions for Property Type and Energy Efficiency Rating: Positive values indicate higher damp risk, negative values indicate lower risk, and larger magnitudes reflect stronger predictive impact.

#### *Construction age*

In Fig. 3, older homes were more likely to be classified as damp, whereas newer homes showed lower predicted damp risk. This trend was further reinforced in Fig. 4, where SHAP values highlighted shifts in damp risk that aligned with historical changes in construction materials, insulation practices, and regulatory standards.

Both construction norms and formal standards have influenced construction practices over time. For instance, the shift from solid to cavity walls in the 1920s initially occurred as an industry-driven norm before becoming a regulatory standard with the 1965 Building Regulations, which mandated cavity walls in new constructions. Similarly, while insulation in cavity walls was widely adopted in the 1970s due to energy efficiency concerns^31^, it was not legally required until the 1990s. These transitions appear reflected in the SHAP values (Fig. 4), illustrating the influence of evolving construction practices and formal standards on damp risk over time.

A Chi-Square test (*p* = 4.70 × 10⁻²⁰) confirmed a significant association between Construction Age Band and Wall Type, showing distinct structural practices across periods. In the data, Pre-1930 homes were predominantly solid brick walls (70%), with 57.6% uninsulated and 12.1% externally insulated, aligning with higher SHAP values for damp risk (Fig. 4). For homes built between 1967 and 1990, SHAP values indicated a rise in damp risk, with homes containing 63% retrofitted cavity walls and a mix of as-built insulated (8.5%), uninsulated (8.5%), and externally insulated (6.7%) cavity walls. These finding suggest that damp issues were present in both retrofitted and newly built cavity walls. Post-1990, SHAP values stabilized, reflecting a time of improved insulation standards and stricter regulations, with mandatory insulation from the 1990s and accredited construction details introduced by 2010. While these changes coincide with a decline in damp risk, vulnerabilities persist across all construction age bands, highlighting the importance of proper installation, ventilation, and moisture management.

To further investigate how Construction Age Band and other variables influence Wall Energy Efficiency, Kruskal-Wallis tests were applied, allowing comparisons across multiple independent groups. The results showed significant differences in Wall Energy Efficiency across Construction Age Band (*p* = 9.02 × 10⁻⁶⁵) and Wall Type (*p* = 1.56 × 10⁻⁹²), confirming that insulation quality varies by construction period and wall type. This test was chosen because Wall Energy Efficiency is an ordinal variable, meaning it follows a ranked order without assuming a normal distribution. However, while the Kruskal-Wallis test detects group differences, it does not measure their strength, direction, or causality.

Since Construction Age Band varies depending on Wall Energy Efficiency, a logistic regression model was applied to assess whether construction period directly influences damp risk while controlling for multiple factors. The results showed that Construction Age had no significant direct effect on damp risk (*p* > 0.97), indicating that older homes were not inherently more prone to damp once Wall Energy Efficiency and other factors were considered. However, Wall Energy Efficiency remained a significant predictor (*p* = 0.0339), reinforcing its role in damp occurrence. The non-significant interaction terms (*p* > 0.08) suggest that the impact of Wall Energy Efficiency on damp risk does not vary across construction periods, meaning that insulation performance is a more critical factor than construction age alone.

#### *Other factors*

Among the other variables, the Mann-Whitney U test on SHAP values did not find a statistically significant difference between damp and non-damp groups, suggesting a weak individual effect. However, when these variables were removed and the model was retrained, performance declined, indicating that while they may not show significance individually, they contribute to the model through interactions with other features, ultimately improving damp prediction accuracy.

It was noted however that both “Property type” and “Energy Efficiency Rating” displayed interesting behaviour which may warrant further study (Fig. 5). Flats tend to result in positive damp predictions, whereas bungalows result in lower predictions. This may be due to limited ventilation in flats, as they have fewer external walls for windows or extraction systems.

The SHAP analysis (Fig. 5) further reveals that energy efficiency ratings generally correlate with lower damp risk, as better-performing properties have lower SHAP scores. However, once the EPC rating reaches “B”, SHAP scores become positive again. The data provides some insight into this pattern as 83% of B-rated properties are flats, and 79% of these have a small floor area of less than 69 square metres. This suggests that while improved insulation in B-rated properties enhances thermal performance, the combination of high airtightness, small living space, and limited ventilation in flats may create conditions where moisture accumulation becomes more likely. This trend also aligns with the SHAP results for property type (Fig. 5), which indicate that flats are more vulnerable to damp, reinforcing the idea that ventilation constraints in highly insulated, compact flats may contribute to increased damp risk.

## Discussion

The results show that Machine Learning does indeed have the capability to predict a home’s risk of damp based on construction details. Metrics such as accuracy, precision, recall, F-measure, and AUC indicated that the random forest provided reliable predictions across different data conditions. This aligns with prior research that highlights the random forest model’s effectiveness in managing complex datasets^32–34^. The study also found that models trained on balanced datasets consistently outperformed those trained on imbalanced datasets, aligning with existing literature that balanced data reduces bias toward the majority class^35,36^. Balanced models in this study demonstrated superior recall and F-measure, effectively identifying positive class cases (damp homes), while maintaining precision. In contrast, imbalanced training led to higher overall accuracy but poor positive class detection, reaffirming the value of balanced datasets for robust and generalizable model performance.

The SHAP analysis provided understanding of the factors influencing damp risk in homes, aligning with existing literature but also revealing some counterintuitive insights. Consistent with previous studies, features such as high modelled heating costs, low energy efficiency, and smaller property size were generally associated with higher damp risk, perhaps due to factors such as poor insulation and limited ventilation which exacerbate moisture retention and lower surface temperatures, increasing RH and promoting mould growth^1,15^. However, the model also suggested some counterintuitive patterns, such as walls with “Average” energy efficiency decreasing risk of damp, whereas “Good” walls increase risk. This highlights the complex nature of damp and the requirement for sophisticated models to account for these nuances when predicting damp risk.

The damp predictive model in this study presents one way by which housing managers could move towards a more proactive damp management strategy. Homes identified as “high-risk” by the algorithm could be prioritized for early surveys and preventative interventions, reducing long-term remediation costs for housing associations while improving occupant health outcomes. Integrating predictive modelling into maintenance workflows could enable housing associations to allocate resources more effectively by scheduling inspections based on risk levels rather than relying solely on resident complaints. Additionally, risk-based models could support strategic planning, helping housing providers identify patterns of damp prevalence across property types and inform targeted investment in retrofitting or insulation upgrades.

Predictive models for damp have potential applications outside of housing associations. For policymakers, predictive models could offer a data-driven approach to housing quality regulation. Local councils and national authorities could use damp risk assessments to guide funding allocations for damp mitigation initiatives, ensuring that high-risk properties receive priority support. Furthermore, integrating damp risk predictions into building standards and energy efficiency policies could enhance regulatory frameworks, ensuring that vulnerable homes receive timely interventions before damp conditions worsen. Landlords, particularly in the private rental sector, could also benefit from predictive modelling by incorporating damp risk predictions into routine property management. This could facilitate compliance with housing quality regulations, such as the Homes (Fitness for Human Habitation) Act 2018, by identifying and addressing damp issues before they escalate into tenant disputes or legal claims. Finally, rental property rating systems could incorporate predictive damp risk scores to improve transparency for prospective tenants, supporting informed decision-making in the housing market.

While this study demonstrates the potential of machine learning for damp risk prediction, further improvements could enhance its predictive capabilities. The random forest, which achieved an accuracy of 0.636 on balanced data and 0.793 on imbalanced data, performed well, but its predictive power remains limited by the available dataset. One key limitation is the absence of occupant-related factors, such as ventilation practices, heating behaviour, and moisture-generating activities, which significantly influence damp formation^37^. Incorporating additional variables, such as historical damp repairs and real-time environmental monitoring sensor data (e.g., temperature and relative humidity), could further improve accuracy by capturing dynamic damp-related conditions. Furthermore, alternative modelling approaches could refine performance. Ensemble learning techniques, such as stacking multiple classifiers or hybrid deep learning models^38^, may improve generalization by capturing complex relationships among damp risk factors. Additionally, feature engineering, such as introducing interaction terms between energy efficiency measures, property type, and wall construction type, may reveal more nuanced risk patterns. Exploring these refinements, along with integrating occupant behaviour data and sensor-based environmental monitoring, could strengthen the model’s reliability and practical applicability, making it a more effective tool for proactive damp management.

Another limitation is that the model’s predictions are probabilistic, estimating the likelihood of damp rather than providing definitive classifications. Indeed, providing a definitive classification of what constitutes a “damp” home in itself presents a challenge – most homes in the UK will have at least a small level of damp and mould, for example around windowsills, and most would not class this level of damp as an issue. Drawing a dividing line on the wide spectrum of damp issues to decide when a home is “damp” or not, is therefore a matter of human judgement, and partial to individual differences. Standardised and robust guidance on when to class a home as having problematic damp could help alleviate this limitation. Such guidance could also allow for the development of a quantitative metric for how damp a home is. Future algorithms could then seek to be more descriptive in their predictions, either predicting this quantitative metric, or perhaps predicting more specific damp classes that describe damp type and/or room (e.g. “Condensation damp – Bedroom”).

In this study the input data was sourced from an individual housing association. Although this housing association owns a diverse range of properties across more than 100 local authorities in England, relying on a single source limits the representativeness of the data. Homes outside of this housing association, such as those which are privately-owned, likely show different damp patterns due to variations in maintenance practices or energy efficiency measures. In particular, 64% of homes in England are estimated to be owner-occupied, with these properties tending to be larger and consisting of more houses instead of flats^39^. This restriction reduces the ability of the model to capture the full spectrum of damp-related issues in English homes. Future studies could train models using datasets from diverse geographic regions and housing contexts to improve the generalizability of damp prediction models. Additionally, validating the model on independent datasets from different housing providers, local authorities, and socio-economic settings would provide a more comprehensive assessment of its predictive reliability and robustness.

A machine learning model is only as good as its input data. This study relied on data from the EPC database. While EPC data has the benefit of being easily accessible, it is limited to only describing the building. Furthermore, it has established issues with data quality^40^. Future research directions should therefore consider incorporating more diverse and comprehensive input for prediction. For example, including climate data, information on building materials and socio-demographic information could provide more nuanced predictions. Longitudinal studies that track changes in damp risk over time could also provide insights into how factors like property maintenance, renovations, and climate change influence damp in homes. Additionally, expanding the use of SHAP and other interpretability methods could further improve the practical utility of machine learning models for housing studies, allowing for the translation of research findings into actionable policy recommendations and interventions.

A final and important factor not considered in this work is ventilation. EPC records include only limited information on this, assigning each home into a broad ventilation category. In these data, almost all homes were assigned to the “natural ventilation” category. As a result, this variable was excluded from the machine learning model. However, previous studies have highlighted that airtight homes retain humidity, increasing the risk of condensation^30^. Using machine learning to study the interplay between insulation, ventilation and damp could therefore provide valuable information into how to prevent future damp cases. Likewise, the “heating cost” in an EPC is a modelled cost to achieve a standardised heating schedule, rather than reflecting the true energy consumed. The observed relationship between “heating cost” and damp risk may therefore reflect the impact of insufficient heating rather than the direct effects of an energy inefficient house. Additional data on true energy consumption would likely improve any models considerably. Further research is therefore needed to explore how heating behaviours interact with energy efficiency in shaping damp risk, particularly in homes where affordability limits heating use.

## Conclusion

This study demonstrates the potential of supervised machine learning to predict damp issues in residential buildings using real-world data. After testing seven machine learning algorithms; neural networks, decision trees, XGBoost, random forest, support vector machines, logistic regression, and K-nearest neighbors, the random forest emerged as a strong algorithm to create such a prediction. It achieved an accuracy of 0.636 on balanced data and 0.793 on imbalanced data, demonstrating robust performance across both datasets. The model was trained on data from up to 2,073 homes, using housing inspection records to identify cases of damp, and supplementary information from the national Energy Performance Certificate (EPC) database. SHAP analysis highlighted key predictors of damp, including heating costs, energy efficiency, and wall efficiency, offering insights into the factors which are most useful when assessing damp risk.

Using machine learning to predict damp could mark a shift from traditional, reactive approaches that rely on physical surveys and remediation once damage has already occurred. Indeed, recent UK guidance calls for proactive damp management strategies to be implemented across the sector^9^. Machine learning may offer a proactive solution by identifying homes at risk of damp and facilitating preventative measures before damp escalates into serious issues.

Overall, the results of this work suggest that such proactive damp management could be implemented with relative ease. The input data used in this work was sourced from EPCs – a data source which housing associations and local councils will have easy access too. It would therefore be straightforward for these organisations to create their own algorithm or apply the presented algorithm to their own data. Homes could then be prioritised for survey and early intervention, with new data being ingested into the algorithm to allow continual learning. EPC data is, however, very limited and describes only the building fabric and heating/hot water system. Incorporation of additional data on weather, actual energy consumption, occupancy patterns and socio-demographic factors would be highly likely to improve the accuracy of such algorithms considerably. Therefore, the use of machine learning in assessing damp risk in residential properties is an area which warrants further attention, with future research focusing on enhancing the model’s performance, incorporating additional datasets, and exploring advanced machine learning techniques for more comprehensive damp prediction.

## Methods

The implementation of machine learning in this study followed a multi-step process, beginning with data preparation and continuing through model training, evaluation, and SHAP analysis. The aim was to develop a predictive model for damp in residential buildings using machine learning techniques.

### Data collection and preprocessing

This study explores the use of supervised machine learning to predict damp in residential properties. The dataset evolved in two stages. Initially, the study used a balanced dataset that was naturally created from the inspection data made available from a UK housing association. In September 2021, a housing association provided initial data on 945 homes inspected for Housing Health and Safety Rating System (HHSRS) hazards, including damp. Damp cases were identified explicitly, while non-damp homes remained in the dataset if they had other recorded housing hazards but no mention of damp. Additional information was then incorporated by linking the dataset with the Energy Performance Certificate (EPC) database using address-based matching. As EPCs have been mandatory since 2008 for all newly built, sold, or rented properties, they provided easily accessible insights into energy performance and building features, enriching the dataset for damp prediction^41^. After data cleaning and EPC matching, homes without corresponding EPC records were excluded, resulting in a refined dataset of 869 homes, with 426 classified as “Has Damp” and 443 as “Non-Damp”. This dataset naturally achieved near-equal representation of damp and non-damp homes, reflecting the distribution of damp occurrences within the sampled properties at that stage. It covered 50 local authorities, with Huntingdonshire contributing the largest share (57.4%), followed by Chorley, Sheffield, and other areas.

As additional data became available, the dataset expanded, leading to a shift toward an imbalanced dataset with more damp homes than non-damp homes. In March 2022, the housing association provided data on 368 additional homes, followed by another dataset in October 2022 containing 1,239 homes. These new datasets differed from the initial sample as they were explicitly focused on homes where damp issues had already been reported, inspected by damp specialists following resident complaints. After data cleaning and EPC matching, 1,204 damp homes with complete EPC data were integrated into the dataset. This led to a final imbalanced dataset of 2,073 homes, comprising 1,630 “Has Damp” and 443 “Non-Damp” homes. The dataset then spanned 125 local authorities, with Huntingdonshire continuing to contribute the largest share (31.1%), followed by Chorley, Sheffield, and Preston. Social housing remained predominant (88.9%). Since all additional homes were identified as damp, the non-damp count remained unchanged, naturally creating the imbalance.

It is important to note that the imbalanced dataset contains a higher proportion of damp homes than non-damp homes. This reflects the targeted nature of data collection rather than the general distribution of damp in the housing stock. According to the English Housing Survey, approximately 30% of households, or seven million homes, report damp issues. However, as the dataset was constructed from housing association inspection records, primarily triggered by resident complaints and disrepair claims under the HHSRS, it naturally overrepresents homes where damp was identified as a documented issue rather than occurring across the full housing sector. Thus, while this dataset allows for testing of algorithms on imbalanced data which is likely to be encountered in the real-world, real-world data is likely imbalanced in the other direction.

Class imbalance is a recognised challenge in data mining and machine learning, where models often struggle to accurately predict the minority class due to biases favouring the majority class^42,43^. While techniques such as under-sampling, over-sampling, and synthetic methods like SMOTE are available to address imbalance^43^, this study chose not to artificially modify the dataset to preserve the real-world distribution of damp occurrences and ensure methodological consistency. The natural evolution of the dataset provided both a balanced and an imbalanced dataset, allowing the evaluation of models under different class distributions without introducing synthetic patterns.

Although oversampling and synthetic data generation methods like SMOTE can improve recall for the minority class, they may also introduce artificial feature correlations that do not exist in real housing stock. Given that the dataset already contained a naturally balanced subset, the decision was made to evaluate model performance using real-world imbalanced conditions, which housing associations and policymakers are likely to encounter. However, future research could explore alternative resampling methods, particularly in scenarios where maximizing the sensitivity of damp risk predictions is a priority. Adaptive synthetic sampling techniques or cost-sensitive learning approaches could be investigated to refine the trade-off between recall and precision while maintaining interpretability.

To prepare the data for machine learning algorithms, categorical variables were encoded using one-hot encoding. This method converts categorical variables into binary vectors, ensuring they are appropriately represented for machine learning models without implying ordinal relationships. For numerical features, feature scaling was applied to ensure all variables contributed equally to the algorithms. Standardisation was used to transform numerical features to have a mean of 0 and a standard deviation of 1. This approach prevents differences in value ranges from distorting calculations, particularly in distance-based algorithms such as the support vector machine (SVM) and k-Nearest Neighbours (k-NN)^44^.

### Feature selection

Feature selection is a process in machine learning aimed at enhancing model performance and interpretability by reducing dataset dimensionality. This is accomplished by eliminating irrelevant and redundant features, which can lead to overfitting, increased complexity, and reduced interpretability^45,46^. In this study, the dataset initially consisted of hundreds of observations with 86 independent variables. To optimize model performance and ensure the inclusion of only the most relevant features, a hybrid feature selection approach was employed, combining filter and wrapper methods. For numerical variables, a correlation matrix was first used to identify and remove highly correlated variables, ensuring that only the most independent and relevant features were retained. This step helped to prevent multicollinearity, which could distort the model’s predictions. For categorical variables, Chi-Squared tests and Fisher’s Exact Test (for small sample sizes) were applied to evaluate their statistical significance in relation to the target variable of whether a home has damp or not.

Following the initial filtering, the wrapper method was used to further refine the feature set. Specifically, Recursive Feature Elimination (RFE) with a random forest algorithm was employed to iteratively remove the least important features. RFE assesses feature importance based on the model’s performance and eliminates variables that contribute the least to predictive accuracy.

The hybrid approach also involved strategic adjustments to feature selection. Before applying the filter and wrapper methods, certain variables such as tenure and local authority were excluded based on preliminary data analysis. This exclusion was necessary because these variables showed irregular patterns in how they were distributed across the UK, as well as a disproportionate number of properties being under social rental agreements. Removal of such variables was required to reduce the risk of introducing bias into the model. Additionally, certain variables, although not highlighted as significant by the hybrid approach, were included based on their established importance in related literature and their relevance to damp issues.

The final selected variables for the model are displayed in Table 1:

### Training and testing data split

In supervised machine learning, splitting the dataset into distinct training and testing subsets is a critical step that underpins the model development process. The training set is used to build and train the model by allowing it to learn from the data, while the testing set is reserved to evaluate the model’s performance on unseen data. This separation is essential for assessing how well the model generalizes to new data, which is a fundamental goal in predictive modelling.

In this study, the dataset was split into a 70% training set and a 30% testing set. The separation of the dataset into a 70/30 split ratio was justified by empirical research and best practices in the field of machine learning^47–49^. The 70/30 ratio is widely recognized as an optimal balance for training and testing, particularly in scenarios involving smaller datasets where overfitting poses a significant risk. Random sampling was employed to create the train-test split, increasing the probability that both subsets were representative of the overall dataset.

### Selected algorithms

In this study, a variety of machine learning algorithms were selected based on their effectiveness in handling classification tasks, particularly in predicting the likelihood of damp in homes. These algorithms were chosen due to their frequent use in the literature and for their ability to model complex relationships and handle both categorical and numerical data^22,24,42,50–53^. The selection aimed to compare the performance of different models to identify the most accurate and efficient algorithm for damp prediction.

In this study, all machine learning models were implemented using the R programming language. The following algorithms (and their respective packages) were employed; neural networks (“nnet”), decision trees (“rpart”), XGBoost (“xgboost”), random forest (“randomForest”), support vector machines (“e1071”), logistic regression (“caret”), and K-Nearest Neighbors (“caret”).

### Model training, Cross-Validation and hypermeter tuning

To ensure that the machine learning models in this study were generalizable and not overfitted to the training data, a 10-fold cross-validation (CV) technique was employed. This approach was applied to both balanced and imbalanced datasets to assess model performance. Initially, the dataset was divided into a training set and a blind testing set. The blind set was reserved for the final evaluation of model performance, while the training set was split into 10 folds for cross-validation. Cross-validation is a widely recognized method that enhances the robustness of model evaluation by systematically dividing the training data into subsets, or “folds.” In each iteration, the model is trained on a portion of these folds and validated on the remaining fold, repeating the process 10 times to ensure each fold is used for validation once. This process ensures that the model is not overly tuned to one particular subset of the data, which could lead to overfitting^44,54–56^.

Hyperparameter tuning is another critical aspect of model development that directly influences training and predictive outcomes. Unlike model parameters, which are learned during training, hyperparameters are set before training and guide the model’s learning process. Proper tuning of these hyperparameters ensures that the model achieves a balance between complexity and generalizability, again minimising overfitting^57,58^. In this study, grid search analysis was used to optimize the hyperparameters for each machine learning model. Grid search systematically tests multiple combinations of hyperparameter values and evaluates their impact on model performance, which is a computationally intensive but effective method for identifying the best configurations^58^. Each combination of hyperparameters was evaluated during the 10-fold cross-validation process, ensuring that the model was fine-tuned to maximize its predictive performance. The grid search analysis identified the most effective hyperparameters for each model as shown in appendix 1.

### Model performance evaluation

The performance of the machine learning models was evaluated using 10-fold cross-validation, a widely recognized method for assessing model generalizability to unseen data. Key performance metrics, including accuracy, precision, recall (sensitivity), F1-score, and the Area Under the Curve (AUC)^59,60^, were used to comprehensively evaluate predictive capabilities. Each metric ranges from 0 to 1, where higher values indicate better performance. Accuracy measures the overall correctness of predictions, with 1 indicating perfect classification and 0.5 representing performance equivalent to random guessing in a binary classification task. Precision assesses the proportion of predicted damp homes that are actually damp, with higher values indicating fewer false positives. Recall (sensitivity) measures the model’s ability to correctly identify damp homes, where higher values indicate fewer false negatives. F1-score provides a balance between precision and recall, ensuring that both false positives and false negatives are minimized. AUC (Area Under the Curve) evaluates the model’s ability to distinguish between damp and non-damp homes across different decision thresholds, with values closer to 1 indicating stronger discrimination and 0.5 suggesting no distinction beyond random chance.

Cross-validation was applied to all seven machine learning algorithms across both balanced and imbalanced datasets. After the cross-validation process, the final model evaluation was conducted using a reserved blind test set, comprising 30% of the original dataset. This test set was used to determine the models’ ability to generalize to new, unseen data, which is essential for real-world applications. The models were evaluated on this test set using the same performance metrics (accuracy, precision, recall, F1-score, and AUC), allowing for a comparison between cross-validation results and the test set performance. Any significant discrepancies between these two phases could indicate overfitting, where the model performs well on the training data but struggles with new data.

### Analysis of damp contributing factors: SHAP, causal, and statistical approaches

A combination of machine learning explainability, causal inference, and statistical hypothesis testing was used to identify and validate key factors influencing damp risk. Each method was selected to allow an assessment of feature importance, relationships, and predictive reliability. Since machine learning models often lack interpretability, SHAP (SHapley Additive exPlanations) were first calculated to interpret the models’ predictions and provide insights into the most influential features for predicting damp. SHAP values quantify the contribution of each feature to the model’s output, offering both local explanations (how specific features influence individual predictions) and global insights (overarching trends across the dataset). SHAP, rooted in cooperative game theory, is a model-agnostic method that offers consistent and unified explanations, making it ideal for understanding the complex relationships between features and model predictions^61^.

Statistical hypothesis testing was applied to validate a variables significance. In all cases, two-tailed tests were used, along with an α value of 0.05. Before applying statistical tests on continuous data, normality was assessed using the Kolmogorov-Smirnov test with Lilliefors correction^62^. This test evaluates whether the sample distribution significantly deviates from a normal distribution by comparing it to an expected Gaussian distribution. The results indicated significant deviations from normality (*p* < 2.2 × 10⁻¹⁶ for heating cost, *p*= 6.117 × 10⁻⁷ for floor area), suggesting that the data does not follow a normal distribution. Additionally, Q-Q plots and histograms were examined, revealing skewed distributions that further supported non-normality. Given these findings, a non-parametric alternative - the Mann-Whitney U test^63,64^- was applied to ensure valid statistical inference without assuming normality.

The Kruskal-Wallis test^63,64^ was used to compare ordinal variables, such as wall energy efficiency, across multiple construction periods. Ordinal variables have a natural ranking but do not have equal intervals between categories (e.g., “Very Poor” to “Very Good” insulation). Unlike the Mann-Whitney U test, which is limited to comparing two groups, the Kruskal-Wallis test allows for evaluating differences across three or more independent groups without assuming a normal distribution. This made it suitable for assessing whether insulation quality varied significantly between multiple construction periods.

Chi-Square tests^65^ evaluate the association between categorical variables by comparing observed and expected frequencies under the assumption of independence. In this study, it was applied to assess whether the distribution of wall energy efficiency and construction period differs across damp classifications. This non-parametric test is suitable for examining relationships between discrete variables without assuming normality or equal variance.

Logistic regression^66^ is a statistical model used to estimate the probability of a binary outcome based on one or more predictor variables. In this study, it was applied to assess the influence variables such as wall energy efficiency and construction period on damp occurrence while controlling for potential confounders such as heating cost, floor area, and energy consumption. By modelling the relationship between categorical and continuous predictors with a binary outcome, this approach isolates the independent effect of each variable while accounting for other influencing factors.

Analysis of Variance (ANOVA)^63,64^was used to evaluate whether there were significant differences in variables such as floor area and wall energy efficiency across multiple groups. Since ANOVA determines only if a significant difference exists but does not specify between which groups, Tukey’s post-hoc test was applied to identify specific pairwise differences^63,64^. This method controls for multiple comparisons, ensuring statistically valid distinctions between categories.

The Overlap Coefficient (OVL)^67^ was used to quantify the degree of distribution overlap between groups. For example, floor area was assessed to determine whether its distribution meaningfully differs between damp and non-damp homes. A high OVL indicates substantial overlap, suggesting that the variable may not independently distinguish between groups and could be influenced by other interacting factors.

To investigate potential causal mechanisms, Causal Mediation Analysis (CMA)^27^ was applied to explore indirect relationships between variables and their impact on damp occurrence. Unlike SHAP, which provides model interpretability without establishing causality, CMA enables the decomposition of total effects into direct and indirect effects, revealing whether a predictor (e.g., insulation quality) influences the outcome (damp risk) independently or through an intermediary variable (e.g., heating cost). This approach goes beyond simple associations by providing a structured method for evaluating causal pathways. To ensure statistical validity, nonparametric bootstrapping with multiple simulations was employed to estimate confidence intervals for mediation effects. This technique reduces biases associated with parametric assumptions and enhances the robustness of causal inferences. The results offer additional insights into how building characteristics contribute to damp risk through intermediary effects, complementing SHAP-based interpretability with a causal perspective.

## Electronic supplementary material

Below is the link to the electronic supplementary material.

Supplementary Material 1

## Data Availability

The data used in this study were sourced from a UK housing association and the Energy Performance Certificate (EPC) database. Due to confidentiality agreements, the housing association data are not publicly available. However, summary statistics and analysis scripts derived from these data are available from the corresponding author upon reasonable request. The EPC data used in this study are publicly accessible via the Open Data Communities platform at https://epc.opendatacommunities.org/.

## References

[1] Bukky Balogun, F. R. W. Health inequalities: cold or damp homes. *House Commons Libr.* (2023).

[2] WHO. DAMP AND MOULD health risks, prevention and remedial actions. *World Heal Organ.* 1–8 (2009).

[3] Blay, K., Agyekum, K. & Opoku, A. Actions, attitudes and beliefs of occupants in managing dampness in buildings. *Emerald Insight*. **37**, 42–53 (2019).

[4] Burkinshaw, R. How to investigate damp. *How Investigate Damp*. 10.1201/9781003003649 (2020).

[5] Kent, D. Control of Dampness. (2018).

[6] Dell’Isola, M., D’Ambrosio Alfano, F. R., Giovinco, G. & Ianniello, E. Experimental analysis of thermal conductivity for Building materials depending on moisture content. *Int. J. Thermophys.* **33**, 1674–1685 (2012).

[7] All-Party Parliamentary Group for Healthy Homes and Buildings. Building our Future: Laying the Foundations for Healthy Homes and Buildings. White Paper. (2018).

[8] Lee, A., Sinha, I., Boyce, T., Allen, J. & Goldblatt, P. Fuel poverty, and health inequalities in the Uk contents. *Inst. Heal Equity* (2022).

[9] GOV.UK. Understanding and addressing the health risks of damp and mould in the home. 1–55 (2024).

[10] Neil May and Chris Sanders. Moisture in buildings: an integrated approach to risk assessment and guidance. *BSI Stand. Publ* (2017).

[11] WHO. WHO Guidance for indoor air quality: dampness and mould. (2009). 23785740

[12] Franzoni, E. Rising damp removal from historical masonries: A still open challenge. *Constr. Build. Mater.* **54**, 123–136 (2014).

[13] Phillipson, M. C. et al. Moisture measurement in Building materials: an overview of current methods and new approaches. *Build. Serv. Eng. Res. Technol.* **28**, 303–316 (2007).

[14] Nicol, S., Roys, M. & Garrett, H. The cost of poor housing to the NHS. *Build. Res. Establ* 10 (2015).

[15] May, N. & McGilligan, M. U. *Health and Moisture in Buildings - A Report from the UK Centre for Moisture in Buildings*. *UKCMB* (2017).

[16] EHS Stock profile and condition. *Engl. Hous. Surv.* (2017).

[17] GOV.UK. Understanding and addressing the health risks of damp and mould in the home. 1–56 (2023).

[18] UK Government. Damp and mould in social housing Learning the lessons. (2023).

[19] Energy & Industrial Strategy. Energy Follow Up Survey: thermal comfort, damp and ventilation. (2021).

[20] BRE. Fuel poverty. (2017).

[21] EHS. English housing Survey- social rented sector. *Minist Hous. Communities Local. Goverment* **1–73** (2019).

[22] Jordan, M. I. & Mitchell, T. M. Machine learning: trends, perspectives, and prospects. *Sci. (80-)* 349, (2015). 10.1126/science.aaa8415 26185243

[23] Zhang, L. et al. A review of machine learning in Building load prediction. *Appl. Energy*. **285**, 116452 (2021).

[24] Geyer, P. & Singaravel, S. Component-based machine learning for performance prediction in Building design. *Appl. Energy*. **228**, 1439–1453 (2018).

[25] Peng, Y., Rysanek, A., Nagy, Z. & Schlüter, A. Using machine learning techniques for occupancy-prediction-based cooling control in office buildings. *Appl. Energy*. **211**, 1343–1358 (2018).

[26] Perez, H., Tah, J. H. M. & Mosavi, A. Deep learning for detecting Building defects using. *Sensors* **19**, 3556 (2019). 31443244 10.3390/s19163556 PMC6720984

[27] Dustin Tingley, T., Yamamoto, K., Hirose, L. & Keele K. I. mediation: R Package for Causal Mediation Analysis. *JournalofStatisticalSoftware* 59, 23–38 (2014).

[28] Recart, C. & Sturts Dossick, C. Hygrothermal behavior of post-retrofit housing: A review of the impacts of the energy efficiency upgrade strategies. *Energy Build.* **262**, (2022).

[29] Scivyer, B. R. and C. Good Building guide Building damp-free cavity walls. *BRE* (2015).

[30] Brambilla, A. & Sangiorgio, A. Mould growth in energy efficient buildings: causes, health implications and strategies to mitigate the risk. *Renew. Sustain. Energy Rev.* **132**, 110093 (2020).

[31] Historic England. Energy efficiency and historic buildings: insulating early cavity walls. *Hist. Engl.* (2012).

[32] Breiman, L. E. *O Random Forests* 5–32 (2001).

[33] Biau, G. & Scornet, E. A random forest guided tour. *TEST* **25**, 197–227 (2016).

[34] Probst, P. & Wright, M. N. Hyperparameters and tuning strategies for random forest. 1–15 (2019). 10.1002/widm.1301

[35] Ganganwar, V. An overview of classification algorithms for imbalanced datasets. (2017).

[36] Krawczyk, B. Learning from imbalanced data: open challenges and future directions. *Prog Artif. Intell.* **5**, 221–232 (2016).

[37] BS 5250. Management of moisture in buildings — Code of practice. (2021).

[38] RE, M. & VALENTINI, G. *Ensemble Methods*. (2012). 10.1201/b11822-34

[39] EHS. English Housing Survey Headline Report. *Dep. Levelling Up, Hous. Communities* (2022). (2022)-23.

[40] Hardy, A. & Glew, D. An analysis of errors in the energy performance certi Fi Cate database. *Energy Policy*. **129**, 1168–1178 (2019).

[41] EHS Headline Report. English housing survey headline report 2018 to 2019. *Minist Hous. Communities Local. Gov.* 1–59 (2019).

[42] Zemariam, A. B. et al. Employing supervised machine learning algorithms for classification and prediction of anemia among youth girls in Ethiopia. *Sci. Rep.* 1–17. 10.1038/s41598-024-60027-4 (2024). 10.1038/s41598-024-60027-4 PMC11032364 38643324

[43] He, H. & Garcia, E. A. Learning from imbalanced data. *IEEE Trans. Knowl. Data Eng.* **21**, 1263–1284 (2009).

[44] Xu, Y. & Goodacre, R. On splitting training and validation set: A comparative study of Cross – validation, bootstrap and systematic sampling for estimating the generalization performance of supervised learning. *J. Anal. Test.* **2**, 249–262 (2018). 30842888 10.1007/s41664-018-0068-2 PMC6373628

[45] Theng, D. & Bhoyar, K. K. *Feature selection techniques for machine learning: a survey of more than two decades of research*Springer London,. (2024).

[46] Li, J., Cheng, K., Wang, S. & Morstatter, F. Feature Selection: A Data Perspective. 50, (2017).

[47] Nguyen, Q. H. et al. Influence of Data Splitting on Performance of Machine Learning Models in Prediction of Shear Strength of Soil. (2021). (2021).

[48] Verma, C. & Illes, Z. The 15 th International Scientific Conference eLearning and Software for Education Attitude Prediction towards ICT and Mobile Technology for the Real-Time: An Experimental Study using Machine Learning. 12753 (2019).

[49] Muraina, I. O. & IDEAL DATASET SPLITTING RATIOS IN MACHINE LEARNING ALGORITHMS: (2022).

[50] Chakraborty, D. & Elzarka, H. Advanced machine learning techniques for Building performance simulation: a comparative analysis. *J. Build. Perform. Simul.* **12**, 193–207 (2019).

[51] Sarker, I. H. Machine learning: algorithms, Real – World applications and research directions. *SN Comput. Sci.* **2**, 1–21 (2021). 10.1007/s42979-021-00592-x PMC7983091 33778771

[52] Osisanwo, F. Y. et al. *Supervised Mach. Learn. Algorithms: Classif. Comparison* **48**, 128–138 (2017).

[53] Uddin, S., Khan, A., Hossain, M. E. & Moni, M. A. Comparing different supervised machine learning algorithms for disease prediction. *BMC Med. Inf. Decis. Mak.* **19**, 1–16 (2019). 10.1186/s12911-019-1004-8 PMC6925840 31864346

[54] Nti, I. K. Performance of machine learning algorithms with different K values in K-fold Cross- validation. (2021). 10.5815/ijitcs.2021.06.05

[55] Xiong, Z. et al. Evaluating explorative prediction power of machine learning algorithms for materials discovery using K -fold forward cross-validation. *Comput. Mater. Sci.* **171**, 109203 (2020).

[56] Nematzadeh, Z., Ibrahim, R. & Selamat, A. Comparative Studies on Breast Cancer Machine Learning Techniques. *10th Asian Control Conf.* 1–6 (2015) (2015). 10.1109/ASCC.2015.7244654

[57] Pannakkong, W., Thiwa-anont, K., Singthong, K., Parthanadee, P. & Buddhakulsomsiri, J. Hyperparameter Tuning of Machine Learning Algorithms Using Response Surface Methodology: A Case Study of ANN, SVM, and DBN. (2022). (2022).

[58] Bergstra, J. & Bengio, Y. Random Search for Hyper-Parameter Optimization. 13, 281–305 (2012).

[59] Debal, D. A. & Sitote, T. M. Chronic kidney disease prediction using machine learning techniques. *J. Big Data*. 10.1186/s40537-022-00657-5 (2022).

[60] Vujović, Ž. Đ. Classification Model Evaluation Metrics. 12, 6–13 (2021).

[61] Lundberg, S. M. & Lee, S. A unified approach to interpreting model predictions. 1–10 (2017).

[62] Habibzadeh, F. Data distribution: normal or abnormal?? *J. Korean Med. Sci.* **39**, 1–8 (2024). 10.3346/jkms.2024.39.e35 PMC10803211 38258367

[63] Hazra, A. & Gogtay, N. Biostatistics series module 3: comparing groups: numerical variables. *Indian J. Dermatol.* **61**, 251–260 (2016). 27293244 10.4103/0019-5154.182416 PMC4885176

[64] Ajee, K. L., Valsan, A. & Sankaran, R. Choosing the right statistical test: A guide for data analysis. *Amrita J. Med.* **20**, 86–88 (2024).

[65] Franke, T. M., Ho, T. & Christie, C. A. The Chi-Square test: often used and more often misinterpreted. *Am. J. Eval*. **33**, 448–458 (2012).

[66] Maalouf, M. Logistic regression in data analysis: an overview. *Int. J. Data Anal. Tech. Strateg*. **3**, 281–299 (2011).

[67] Martínez-Camblor, P. About the use of the overlap coefficient in the binary classification context. *Commun. Stat. - Theory Methods*. **52**, 6767–6777 (2023).

