# Supplementary material for: A predictive model for damp risk in english housing with explainable AI
Source: Sci Rep. 2025 Apr 12;15:12658. doi: 10.1038/s41598-025-96396-7 (PMC11993753; doi:10.1038/s41598-025-96396-7)
Supplement: Supplementary file 1 — Supplementary Material 1 [file 41598_2025_96396_MOESM1_ESM.pdf]

Appendix 1: Hyperparameter Configurations

This appendix provides details of the hyperparameter configurations for the machine learning models used in the study. These configurations were optimized through a grid search process conducted during 10-fold cross-validation to achieve the best performance.

Model	Optimal Hyperparameter	Explanation
1. Neural Network	size = 3, decay = 0.1	- size: Number of neurons in the hidden layer. A larger size increases model complexity.<br>- decay: Regularization parameter that penalizes large weights to avoid overfitting. Lower decay allows more complex models, higher decay simplifies the model.
2. Decision Tree	cp = 0.01	- cp: Complexity parameter that controls the size of the decision tree. Lower cp results in a larger tree with more splits, while higher cp prunes the tree to prevent overfitting.
3. XGBoost	nrounds = 100, max_depth = 7, eta = 0.3, gamma = 0, colsample_bytree = 0.7, min_child_weight = 1, subsample = 0.7	- nrounds: Number of boosting iterations. More rounds typically improve performance but can lead to overfitting.<br>- max_depth: Maximum depth of the tree. Controls model complexity; deeper trees capture more interactions.<br>- eta: Learning rate, which controls how much each tree contributes to the model. Lower eta results in more robust models but requires more boosting rounds.<br>- gamma: Minimum loss reduction to make a further partition. Controls tree growth and regularization.<br>- colsample_bytree: Fraction of features to sample for each tree. Helps in reducing overfitting by introducing randomness.<br>- min_child_weight: Minimum sum of instance weight needed in a child. Controls tree depth and helps prevent overfitting.<br>- subsample: Fraction of observations to use for fitting each tree. Reduces overfitting by introducing randomness.
4. Random Forest	mtry = 7 ntree = 500, min.node.size = 3, splitrule = "gini", sampsize = 422 (0.75% data)	- mtry: Number of features randomly sampled at each split. A higher mtry may lead to better models at the risk of overfitting, while a lower mtry helps to reduce overfitting.<br>- ntree: Number of trees in the forest. More trees increase model stability.<br>- min.node.size: Minimum size of terminal nodes. Controls the size of the nodes; smaller values lead to deeper trees and more complex models.<br>- splitrule: Criterion used for splitting (gini or entropy). Controls how the splits are made in each tree.<br>- sampsize: Fraction of samples to draw for training each tree. Affects the randomness and robustness of the model.
5. Support Vector Machine	C = 10, sigma = 0. 1	- C: Regularization parameter. A higher C value allows less margin violation but can overfit. Lower C values create a wider margin but may misclassify more points.<br>- sigma: Width of the Gaussian (RBF) kernel. Controls the influence of each support vector, affecting the model's ability to fit the data.
6. Logistic Regression	alpha = 0.5, lambda = 0.001	- alpha: Elasticnet mixing parameter. Balances between L1 (lasso) and L2 (ridge) regularization.<br>- lambda: Regularization strength. Controls the penalty on the magnitude of the coefficients, affecting model complexity and overfitting.
7. K-Nearest Neighbors	k = 13	- k: Number of nearest neighbors to consider when classifying a point. Lower k values make the model more sensitive to noise, while higher k values smooth out predictions by considering more neighbors.
